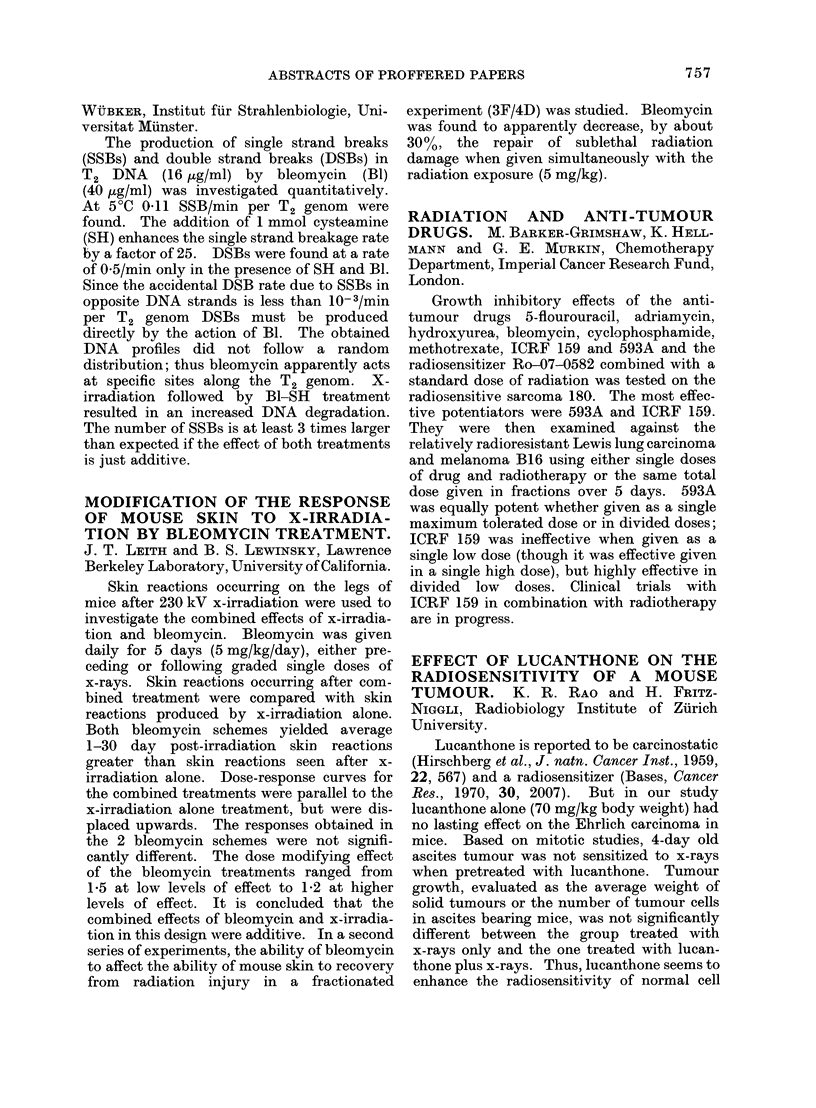# Proceedings: Modification of the response of mouse skin to x-irradiation by bleomycin treatment.

**DOI:** 10.1038/bjc.1975.308

**Published:** 1975-12

**Authors:** J. T. Leith, B. S. Lewinsky


### MODIFICATION OF THE RESPONSE OF MOUSE SKIN TO X-IRRADIATION BY BLEOMYCIN TREATMENT.

J. T. LEITH and B. S. LEWINSKY, Lawrence Berkeley Laboratory, University of California.

Skin reactions occurring on the legs of mice after 230 kV x-irradiation were used to investigate the combined effects of x-irradiation and bleomycin. Bleomycin was given daily for 5 days (5 mg/kg/day), either preceding or following graded single doses of x-rays. Skin reactions occurring after combined treatment were compared with skin reactions produced by x-irradiation alone. Both bleomycin schemes yielded average 1–30 day post-irradiation skin reactions greater than skin reactions seen after x-irradiation alone. Dose-response curves for the combined treatments were parallel to the x-irradiation alone treatment, but were displaced upwards. The responses obtained in the 2 bleomycin schemes were not significantly different. The dose modifying effect of the bleomycin treatments ranged from 1·5 at low levels of effect to 1·2 at higher levels of effect. It is concluded that the combined effects of bleomycin and x-irradiation in this design were additive. In a second series of experiments, the ability of bleomycin to affect the ability of mouse skin to recovery from radiation injury in a fractionated experiment (3F/4D) was studied. Bleomycin was found to apparently decrease, by about 30%, the repair of sublethal radiation damage when given simultaneously with the radiation exposure (5 mg/kg).